# Rapid and Highly Efficient Genetic Transformation and Application of Interleukin-17B Expressed in Duckweed as Mucosal Vaccine Adjuvant

**DOI:** 10.3390/biom12121881

**Published:** 2022-12-15

**Authors:** Xiao Tan, Shuang Chen, Yang Fang, Penghui Liu, Zhubin Hu, Yanling Jin, Zhuolin Yi, Kaize He, Xing Li, Leyi Zhao, Hongning Wang, Hai Zhao

**Affiliations:** 1 CAS Key Laboratory of Environmental and Applied Microbiology, Environmental Microbiology Key Laboratory of Sichuan Province, Chengdu Institute of Biology, Chinese Academy of Sciences, Chengdu 610041, China; 2 Animal Disease Prevention and Food Safety Key Laboratory of Sichuan Province, Key Laboratory of Bio-Resources and Eco-Environment, Ministry of Education, College of Life Science, Sichuan University, Chengdu 610064, China; 3 University of Chinese Academy of Sciences, Beijing 100049, China; 4 Center for Natural Products Research, Chengdu Institute of Biology, Chinese Academy of Sciences, Chengdu 610041, China; 5 Pitzer College, Claremont, CA 91711, USA

**Keywords:** duckweed, *Lemna minor*, interleukin-17B, adjuvant, IBV, transgenic plants

## Abstract

Molecular farming utilizes plants as a platform for producing recombinant biopharmaceuticals. Duckweed, the smallest and fastest growing aquatic plant, is a promising candidate for molecular farming. However, the efficiency of current transformation methods is generally not high in duckweed. Here, we developed a fast and efficient transformation procedure in *Lemna minor* ZH0403, requiring 7–8 weeks from screening calluses to transgenic plants with a stable transformation efficiency of 88% at the DNA level and 86% at the protein level. We then used this transformation system to produce chicken interleukin-17B (chIL-17B). The plant-produced chIL-17B activated the NF-κB pathway, JAK-STAT pathway, and their downstream cytokines in DF-1 cells. Furthermore, we administrated chIL-17B transgenic duckweed orally as an immunoadjuvant with mucosal vaccine against infectious bronchitis virus (IBV) in chickens. Both IBV-specific antibody titer and the concentration of secretory immunoglobulin A (sIgA) were significantly higher in the group fed with chIL-17B transgenic plant. This indicates that the duckweed-produced chIL-17B enhanced the humoral and mucosal immune responses. Moreover, chickens fed with chIL-17B transgenic plant demonstrated the lowest viral loads in different tissues among all groups. Our work suggests that cytokines are a promising adjuvant for mucosal vaccination through the oral route. Our work also demonstrates the potential of duckweed in molecular farming.

## 1. Introduction

Molecular farming that utilizes plants as a platform for production of recombinant biopharmaceuticals has been proposed for more than 30 years [1,2]. Currently, using edible plants has been extensively explored for direct oral delivery of biopharmaceuticals, such as vaccines, antibodies, and other therapeutic proteins [3]. Recombinant proteins expressed in edible plants can be orally administrated, and the plant cell wall can protect them to maintain bioactivity during their delivery to the gut where they are released [4]. Thus, it is possible to skip complex downstream manufacturing processes and this method is very cost effective. Many different plants, including tobacco, lettuce, rice, alfalfa, etc., have been studied for molecular farming [5].

Duckweed is the smallest and fastest-growing angiosperm on Earth [6]. These features make duckweed a well-known model for studying plant biology [7], for example, nucleic acid and protein turnover [8,9], biosynthesis of auxin [10], and photoperiodic control of flowering [11].Researchers have discovered new advantages of duckweed, such as being suitable for exogenous protein expression and able to achieve high protein content under specific cultivation conditions [12,13,14,15]. These advantages, coupled with its fast accumulation rate (biomass doubles under 2 days), make duckweed attractive for the production of valuable therapeutic proteins, such as monoclonal antibodies [16], vaccines [17,18,19], and interferons [13]. In addition, duckweed can be cultivated in a contained system, preventing accidental release of genetically modified plants into the environment, and thus overcomes a serious limitation of commercializing plant expression systems [20]. Therefore, duckweed is a promising candidate for ”molecular farming”. An efficient transformation protocol is essential for developing a plant expression system. The first transient gene expression system in duckweed was reported in 1991 with *Lemna gibba* fronds using gene gun bombardment [21]. The stable transformation system was reported in 1999 in fronds and calluses [22,23]. Up to now, transformation systems have been already established at least seven species, including *Lemna gibba*, *Lemna minor*, *Landoltia punctata*, *Wolffia arrhiza*, *Lemna turionifera*, *Wolffia globosa* and *Lemna aequinoctialis* [13,24,25]. Nevertheless, the high inter- and intraspecific variability makes it difficult to standardize the transformation protocol [7]. Therefore, it is very important to perform optimization for different species, especially on callus induction and transformation conditions [25,26,27].

Vaccines administrated at mucosal sites are called mucosal vaccines. In contrast to injected vaccines, which only induce systemic response [28], mucosal vaccines provoke both systemic and mucosal immune responses, which provide the first line of defense against pathogens that enter the body at a mucosal site [29]. Thus, mucosal vaccination is a better strategy against mucosa-associated infections, including respiratory and gastrointestinal infections [29]. That being said, mucosal vaccines face the challenge of poor immunogenicity. However, alum, the most common adjuvant used in vaccines, is a poor inducer of mucosal immunity [30]. Additionally, bacterial toxins and derivatives, such as cholera toxin (CT) and *Escherichia coli* heat labile enterotoxin (LT), while considered to be efficient for mucosal vaccination, are not safe enough because of their toxicity [30]. Therefore, innovative adjuvants that are safe and strong are in great need for the development of mucosal vaccines. Cytokines play a pivotal role in controlling inflammation and the immune response [31]. Moreover, as endogenous molecules, cytokines are much safer than bacterial toxins and derivatives. Therefore, cytokines are considered to be safe and strong as adjuvants for vaccines, including mucosal vaccines [32]. According to previous reports, different types of cytokines, such as interleukins [33], interferon [34], and TNF-α [35] have been investigated for mucosal vaccines against respiratory tract disease. Interleukin-17B (IL-17B), a member of the IL-17 family, plays a key role in regulating the expression of proinflammatory cytokines. Chicken chIL-17B produced from *Lactobacillus plantarum* NC8 has been reported to enhance chicken’s immune responses to the oral IBV attenuated vaccine, exhibiting potential adjuvanticity on mucosal vaccination [36]. 

Here, we describe how we obtained an efficient transformation system in *L. minor* duckweed and its application in molecular farming. We expressed chIL-17B in *L. minor* and evaluated the biological activity of recombinant chIL-17B in vitro and its immunoadjuvant activities in chickens. We chose the mucosal vaccine against infectious bronchitis virus (IBV), which brings drastic economic loss to the poultry and broiler industry by affecting the production and quality of eggs [37], to evaluate its immunoadjuvant activities. Oral delivery of therapeutic proteins and vaccines in edible plants has been extensively studied, but we evaluate edible plants expressing cytokines as vaccine adjuvants for the first time. We demonstrate that duckweed is a promising platform for molecular farming and chIL-17B expressed in duckweed is an efficient adjuvant for mucosal vaccines.

## 2. Materials and Methods

### 2.1. Plant Materials

*Lemna minor* (*L. minor*) strain ZH0403 (originally named M0157) was provided by the National Aquatic Biological Resource Center (NABRC) (NABRC code for ZH0403: DKLe0329) stored in Chengdu Institute of Biology, Chinese Academy of Sciences (Chengdu, China), and can be obtained by contact with Hai Zhao (zhaohai@cib.ac.cn, Chengdu Institute of Biology, Chinese Academy of Sciences, Chengdu 610041, China). It was cultured on Murashige and Skoog(MS) medium [38], supplemented with 3% sucrose (*w*/*v*) at pH 5.6, and grown at 25 °C with approximately 40 μmol/ m^2^/s 16 h day/8 h night photoperiod.

### 2.2. Callus Induction

The explants of *L. minor* ZH0403 were inoculated on MS, Schenk and Hildebrandt (SH), and Gamborg’s B5(B5) [39] medium and were supplemented with various phytohormone concentrations under different culture conditions (Table 1). Plants were placed on plates filled with medium for callus induction. The induced calluses were then transferred to fresh medium every 2 weeks for future use.

### 2.3. Agrobacterium-Mediated Transformation and Regeneration of L. minor

pCAMBIA2301(p2301), containing the reporter marker gene GUS, was used in the genetic transformation of duckweed. The plasmid vector was introduced into the *Agrobacterium tumefaciens* strain GV3101 by incubating the mixture of plasmid and GV3101 in an ice water bath for 5 min, in liquid nitrogen for 5 min, then at 37 °C for 5 min, and finally in an ice water bath for 5 min. The mixture was grown at 28 °C under shaking condition (150 rpm) overnight in liquid LB medium that contained 50 mg/L kanamycin and 25 mg/L rifampicin. The bacteria were collected at an approximate OD_600_ of 0.6. The sample was then centrifuged at 5000 rpm for 10 min and suspended in MS medium containing 0.6 M mannitol and 100 mM acetosyringone (AS) so that the OD_600_ increased to 1.2. It was then incubated for 1 h at 25 °C. 3 g of the callus was immersed in GV3101 suspension for 10 min and then co-cultured on a medium (MS basal salts, 100 mM AS, 0.2 μM 2,4-D and 4.5 μM 6-BA) for 3 d in the dark in the growth chamber at 25 °C.

After 3 d of co-cultivation, the infected calluses were transferred to solid MS medium (0.2 μM 2,4-D, 4.5 μM 6-BA, 100 mg/L G418 and 200 mg/L Cefotaxime, 3.5 g/L gellan gum, pH 5.6). After 4 weeks of incubation, the light green nodules were collected and stained to monitor the GUS expression. The calluses were transferred into regeneration medium (1/2 SH medium basal salts, 5 g/L sucrose, 100 mg/L G418 and 200 mg/L Cefotaxime). The regenerated fronds were grown on liquid SH medium supplemented with 100 mg/L G418.

### 2.4. PCR Analysis, GUS Staining, and Transformation Efficiency

Total genomic DNA was extracted from both transgenic and wildtype plants following the CTAB method [40]. PCR analysis of putatively transgenic plants amplified *gus* (651 bp) using primers GUS-F: 5′-TGAAGATGCGGACTTACG-3′ and GUS-R: 5′-GTGATGATAATCGGCTGATG-3′. GUS activities in callus and fronds were detected via a GUS histochemical assay kit (Real-Times, Beijing). The stained callus and fronds were observed under a microscope and captured using a digital camera. The transient transformation efficiency, stable transformation efficiency in DNA level, and protein level were calculated using the following formulas:

Transient transformation efficiency = number of calluses expressing GUS/number of total calluses after co-culture [41].

Stable transformation efficiency in DNA level = number of fronds containing GUS gene/number of total calluses after co-culture [42].

Stable transformation efficiency in protein level = number of fronds expressing GUS/number of total calluses after co-culture [43].

### 2.5. Construction of Plant Expression Vectors for chIL-17B

The DNA sequence of chIL-17B was obtained from GenBank (Accession No. XM-015293704.1) and optimized for expression in duckweed via codon usage in *Lemna gibba* (http://www.kazusa.or.jp/codon/, accessed on 9 November 2022). The 5′ end of chIL-17B was linked with a signal sequence in tobacco protein PR1a (GenBank accession no.X06903) by the linker peptide (GSGGS). The 3′ end of chIL-17B was linked with 6× His tags and KDEL sequence. The recombinant gene was named IL-17B and synthesized by the Tsingke Biotechnology Company (Tsingke Biotechnology Company, Chengdu, China). Then the nucleotide sequence of IL-17B was inserted into vector p2301 to replace the β-glucuronidase gene. After sequencing, the resulting plasmid (p2301-IL17B) was transformed into the callus via *A. tumefaciens*-mediated transfection. The resulting calluses were then screened and regenerated. 

### 2.6. Detection and Quantification of the Recombinant Protein

The expression of IL-17B was analyzed using PCR and Western blot assays. Transgenic plants were confirmed via PCR amplification of duckweed genomic DNA using the chIL17B-F: 5′-CCTCCTCTTCCTCGTGAT-3′ and chIL17B-R: 5′-GTCGTGGTTGATGCTGTA-3′ primers. The transgenic and non-transformed (control group) plants were ground in liquid nitrogen and resuspended in PBST, respectively. The solution was centrifuged (12,000 rpm, 20 min, 4 °C) and the resulting supernatant was extracted for Western blot analysis. The extract was placed in a 10% gradient SDS-PAGE and was then transferred onto a nitrocellulose membrane. The recombinant protein was identified using anti-His-Tag monoclonal antibodies (TransGen Biotech, Beijing, China). To determine the expression level of IL-17B in duckweed, various amounts of purified IL-17B proteins from *E. coli* (800, 700, 600, 500, 400, 300, and 200 ng/mL) were analyzed as reference standards via the above Western blot assays. The amounts of IL-17B protein in duckweed were estimated from the values of band intensities using ImageJ [44]. 

### 2.7. Purification of Recombinant chIL-17B

Recombinant IL-17B with a 6× His tag allowed simple purification using Ni-NTA agarose. In accordance with the protocol proposed by Patrycja et al. [45] with minor modification, the transgenic plants (30 g) were homogenized with 3 times of volumes of extraction buffer (1× PBS, 2 mM imidazole, 3% glycerol, 0.2% Tween, pH 7.5). The homogenate was centrifuged three times at 12,000 rpm for 20 min to collect the supernatant, which was then incubated with 3 mL Ni-NTA agarose overnight. On the following day, the agarose was washed with one buffer (1× PBS, 2 mM imidazole, pH 6.8) to remove the untargeted protein and then eluted with another buffer (1× PBS, 250 mM imidazole, pH 6.8) to obtain the targeted protein. The eluted protein was transferred to the third buffer (1× PBS, pH 7.0). The purified protein was stored at −80 °C and used in cell assays.

### 2.8. Analysis of the Bioactivity of cIL-17B in DF-1 Cells

Chicken embryo fibroblast DF-1 cells were cultured in eagle medium (DMEM) (Hyclone Inc., Logan, Utah, USA) containing 10% heat-inactivated fetal bovine serum (Gibco, Grand island, NY, USA) in a humidified 5% CO_2_ incubator at 37 °C. Approximately 1.0 × 10^6^ cells seeded in a six-well plate were stimulated with recombination IL-17B protein (200 ng/mL) for 4 h. Total RNA was extracted from DF-1 cells using Trizol reagent and then reversely transcribed into cDNA by applying a PrimeScript RT Reagent Kit with gDNA Eraser (Takara Bio, Japan). The primers [36] and AceQR qPCR SYBR Green Master Mix (Vazyme Biotech Co., Ltd., Nangjing, China) were used to conduct real-time PCR with Bio-Rad CFX connect (Bio-Rad, Berkeley, CA, USA). All qPCR assays followed the program: 5 mins at 95 °C, followed by 40 cycles of 10 s at 95 °C, and 30 s at 60 °C. GAPDH, the reference gene and the target gene were both amplified. The target gene expression level was calculated via the geometric means method (2^−ΔΔCT^).

### 2.9. Animal Immunization

**Figure 1:**
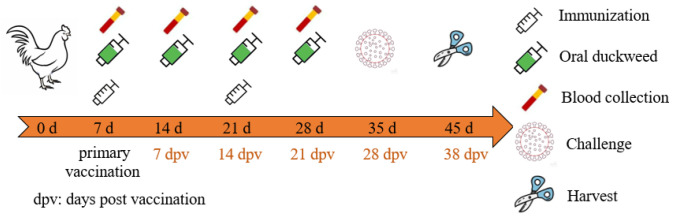
Timeline for vaccination, challenge, blood, and tissue sampling schedules.

Specific-pathogen-free (SPF) White Leghorn chicken eggs (Boehringer Ingelheim Vital Biotechnology Co., Ltd., Beijing, China) were hatched in an automatic incubator (Beijing Haijiang Incubation Equipment Manufacturing Co., Ltd., Beijing, China). After seven days from birth, the chickens were randomly divided into 4 groups with ten chickens each: IL17B adjuvant group (IL-17B-H120), H120 group (PBS-H120), wildtype duckweed control (ZH0403-H120), and PBS control (PBS).ZH0403. As shown in Figure 1, chickens in the IL-17B-H120, ZH0403-H120, and PBS-H120 groups were orally vaccinated with attenuated IBV H120 vaccine (Sichuan HuaPai Bio-Pharmaceutical Co., Ltd., Chengdu, China) before being fed with 200 μL of saline solution containing H120 according to the manufacturer’s instructions on the 7th day for primary vaccination and on the 14th day for booster vaccination. To test adjuvant activity, IL-17B, wildtype duckweed control (ZH0403), and PBS were fed once a week for a four-week period as follows: 0.3 g freeze-dried transgenic duckweed containing about 3 μg chIL-17B protein for the IL-17B-H120 group, 0.3 g non-transgenic duckweed for the ZH0403-H120 group, and 1 mL PBS for the PBS-H120 group. Freeze-dried duckweeds were ground into powder and then suspended in PBS and fed via force feeding through a 2 mL syringe without needles in a volume of 1 mL suspension per chicken. The PBS group chickens were left unvaccinated and infected with IBV. All chickens in the 4 groups were infected with 10^5.8^ EID50 IBV SCMY-19 (200 µL) via the oculo-nasal route after five weeks from birth. The weight of chickens in all groups were recorded weekly until the 28th day post-primary vaccination (dpv) to estimate their growth performance. In addition, there was a negative control group (n = 3) that was neither vaccinated nor challenged with virus. As expected, no virus was detected in this group.

All procedures were approved by the Animal Ethics Committee (AEC) of Animal Experiment Center of Sichuan University (license: SYXK-Chuan-2018-185) and conducted according to the protocols approved by the animal management guidelines of Sichuan University.

### 2.10. Evaluation of the Immunoadjuvant Effect of chIL17B in Chickens

Blood samples of chickens were collected at their wing vein on the 0th, 7th, 14th, 21st, and 28th dpv and then centrifuged twice at 3000 rpm for 10 min to collect the sera. The sera were tested for the IBV-specific antibody titer using the Infectious Bronchitis Virus Antibody Test Kit (IDEXX, Westbrook, ME, USA).

The concentration of IBV-specific sIgA in the lavage fluid was analyzed. The IBV-coated 96-well ELISA plates were incubated with the lavage fluid, then washed and again incubated with goat anti-chicken IgA-HRP (Abcam Ltd., Shanghai, China). Subsequently, the TMB substrate was added to each well for 10 min and then the plates were read at 630 nm on a plate reader.

The tissue samples from trachea, kidney, and lung were collected at 38 dpv and estimated for their viral RNA levels using real-time reverse transcription quantitative polymerase chain reaction (RT-qPCR). Total RNA was extracted from these tissues and converted into cDNA using a Prime Script RT Reagent Kit with gDNA Eraser (Takara Bio, Japan). The virus’ N-gene specific primer pair were as follows: 5′-CGCTCAAGTTCAAGACCTGCTA-3′(forward) and 5′-CATCATCCTGCTTCTTGGCTT-3′(reverse). The amplified product was inserted into the pUC57 plasmid to draw the standard curve.

### 2.11. Statistical Analysis

We analyzed our data through the One-Way-ANOVA test in GraphPad Prism 8. A *p*-value less than 0.05 was considered as significant. 

## 3. Results

### 3.1. An Efficient and Stable Genetic Transformation System for L. minor

**Figure 2:**
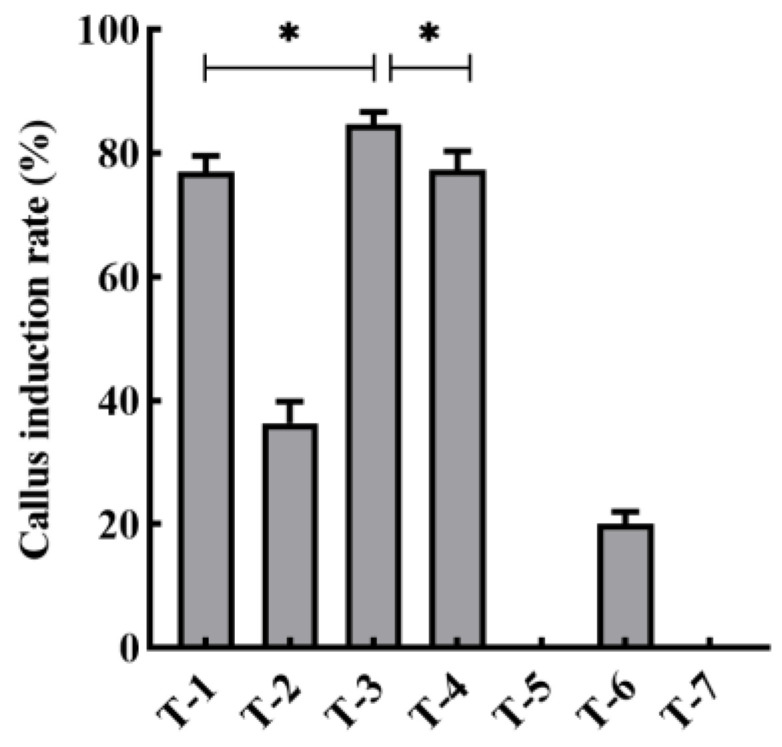
Effect of different mediums and phytohormones on callus induction. T-1 to T-6 denote different methods. The statistical analysis was performed via one-way ANOVA test. Asterisk (*) represents significant difference and corresponds to *p* < 0.05.

**Figure 3:**
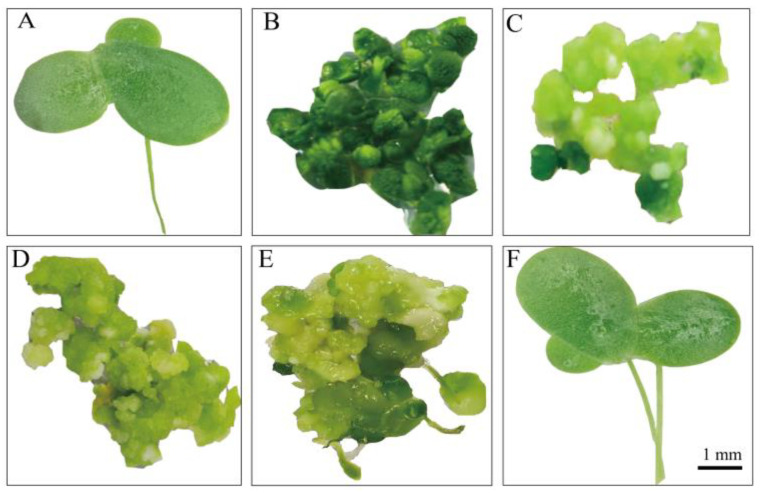
Callus induction and regeneration of *L. minor* ZH0403. (**A**) Frond; (**B**) callus induced from an explant 3 weeks after inoculation; (**C**) callus induced from an explant 6 weeks after inoculation; (**D**) 8-week-old callus; (**E**) 2 weeks of regeneration following (**D**); (**F**) 4 weeks of regeneration following (**D**).

**Figure 4:**
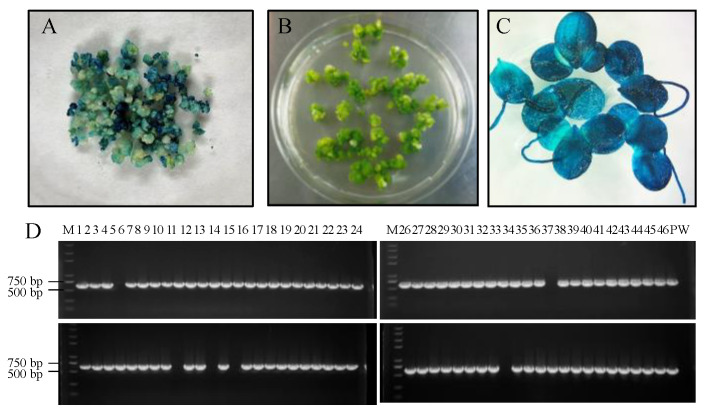
Stable and efficient genetic transformation of *L. minor*. (**A**) GUS-stained co-cultivation of callus and *A. tumefaciens*; (**B**) selected nodule under 100 mg/L G418 stress after 1 month; (**C**) GUS expression in transgenic fronds after 2 months; (**D**) PCR analysis of putatively transgenic plants. M: DNA marker, P: plasmid, W: wild type, 1~92: G418-resistant plants.

An efficient and stable duckweed expression system is the basis for the production of recombinant proteins. We selected different mediums and phytohormones to induce the transformation from fronds to calluses (Table 1). Among these different compositions, T-3 demonstrated the highest callus induction rate of up to 84.6% (Figure 2). Therefore, we used such conditions in the rest of our research. Fronds (*L. minor*) irregularly curled on induction medium after 3 weeks (Figure 3A, B). The curled fronds grew into compact and deep green calluses in the following 3 weeks (Figure 3C). However, when we then transferred the calluses to the subculture medium, they turned into organized light green fragile nodules that were identified as embryonic calluses (Figure 3D) and this step took 2 weeks. The light green nodules were placed on regeneration medium, which then grew fronds and root precursors within only 2 weeks (Figure 3E). After 2 weeks, the precursors turned into normal regeneration plants (Figure 3F). The entire procedure from callus induction to regeneration plants was 12 weeks, including 8 weeks of callus induction and 4 weeks of regeneration plant, which was similar or shorter compared to previous reports [22,46]. To evaluate the transformation rate of calluses, p2301-*gus* was used for genetic transformation. After co-cultivating nodules with A. tumefaciens, more than 95% of the calluses demonstrated GUS activity (Figure 4A), indicating a high overall transient transformation efficiency. The calluses were then transferred to selection medium containing 100 mg/L G418. This concentration of G418 provides an easy way of distinguishing between transformed and untransformed calluses, with the two showing green and white colors, respectively, after 4 weeks. We observed that calluses presented in green more often than presenting in white (Figure 4B). This phenomenon indicated a high screening rate and is correspondent to the infection rate of 95%. Finally, the green calluses were placed on the regeneration medium to produce transgenic fronds for 4 weeks. In order to calculate stable transformation efficiency, we analyzed the incorporation of the *gus* gene at the DNA level and GUS activity at the protein level. We randomly selected 92 independent transgenic lines to detect *gus* via PCR at the DNA level and found that 93% of them demonstrated its presence (Figure 4D). Among the transgenic lines, we discovered that 91% of them displayed GUS activity at the protein level, which means our stable transformation efficiency is 88% at the DNA level and 86% at the protein level. Moreover, the 12 transgenic lines were then cultured for a year and still demonstrated GUS activity (Figure 4C), suggesting long-term stability of the gene’s expression. Together, it takes 7–8 weeks from screening calluses to transgenic plants, a process with high rates of callus infection and screening. 

### 3.2. Expression of Bioactive chIL-17B in L. minor

**Figure 5:**
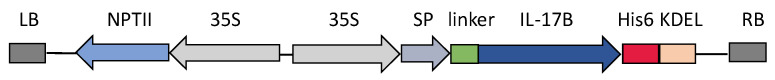
Schematic of T-DNA region of the expression vector construct. Abbreviations: 35S, cauliflower mosaic virus 35S promoter; SP, signal peptide of tobacco-patheogenesis-related 1a protein; His6: histidine tag; KDEL: endoplasmic reticulum retention signal; NPTII: sequence of neomycin phosphotransferase gene.

**Figure 6:**
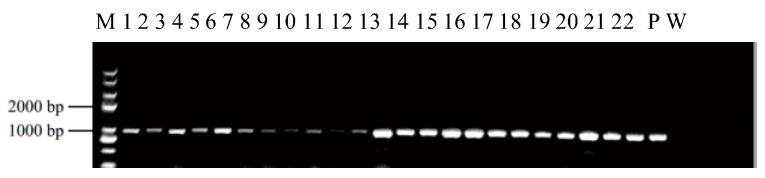
Molecular validation of transgenic lines with 17B specific primers. M: DNA mark, 1~22: transgenic lines, W: wild plant as negative control, P: p2301-17B plasmid as positive control.

**Figure 7:**
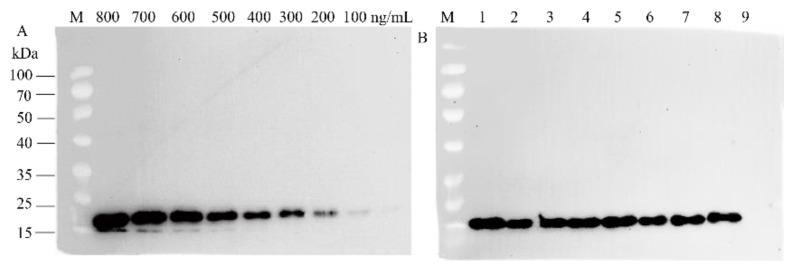
Western blot detection of recombinant protein from transgenic duckweed plants. (**A**) Western blot analysis of various concentrations of standard 17B purified from *E. coli*. (**B**) Western blot analysis of protein samples from plants. M: protein mark, 1–7: transgenic lines, 8: the standard protein (400 ng/mL), 9: wild plant.

To verify the application of our efficient transformation system in *L. minor* as molecular farming, we produced recombinant chIL-17B in our system. The chIL-17B gene was inserted into binary vector p2301, replacing the *gus* gene (Figure 5). To avoid protein degradation and enhance the accumulation of recombinant proteins, we add an endoplasmic reticulum (ER) retention signal KDEL at the C-terminus of recombinant IL-17B, as ER lacks various proteases that regulate post-transcriptional degradation [45]. The vector was then transformed into the callus through *A. tumefaciens*-mediated transfection. Transgenic lines, following infection and regeneration, were obtained and transferred into SH liquid medium containing 100 mg/L G418. The morphological characteristics of transgenic plants demonstrated no difference compared with those of wild type. The IL-17B sequence in G418-resistant plants were amplified using PCR to test the efficiency of incorporating IL-17B gene into *L. minor*. We randomly selected 88 independently transgenic lines, of which 80 contained the target fragment, a screening rate of 90.9%. The same fragment did not appear in wild plants (Figure 6). A Western blot assay was used to confirm the expression of IL-17B in L. minor. The result demonstrated that an expectedly 20.65 kDa protein band was detected using the mouse anti-His-Tag mAb (Figure 7B). Moreover, to quantify the recombinant IL-17B in duckweed, we calculated the band intensities of various concentrations of purified IL-17B proteins from *E. coli* BL21(DE3) based on Western blot assays (Figure 7A). This protein in duckweed is up to 1.89 μg/g FW, corresponding to 0.036% of total soluble protein (TSP). Therefore, we selected the transgenic line 32 with high and stable levels of recombinant protein accumulation to further research. 

**Figure 8:**
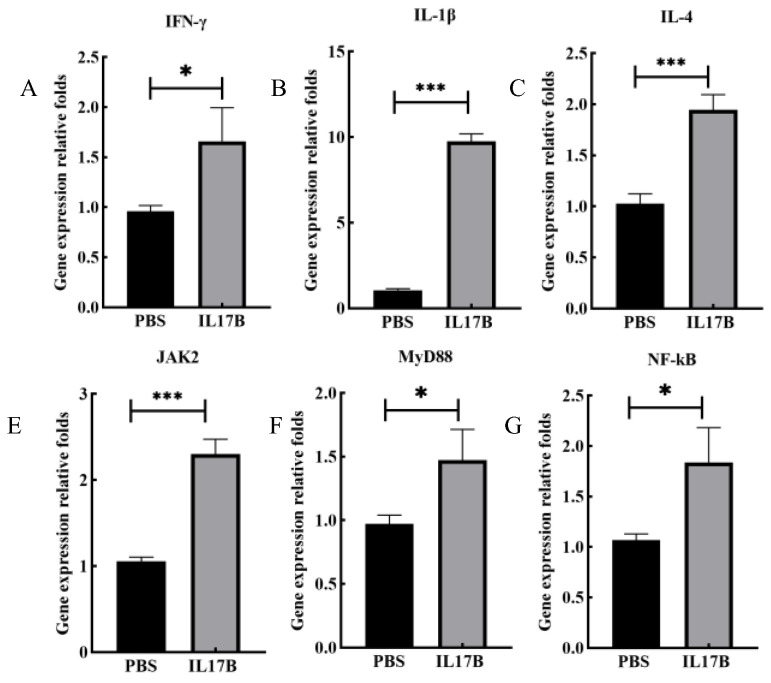
The expression levels of the immune-related genes in DF-1 cells treated using the recombinant protein chIL-17B expressed in duckweed. (**A**–**C**,**E**–**G**)The expression levels of MyD88, NF-κB, IFN-γ, IL-1β, JAK2, and IL-4 in ChIL-17B-stimulated DF-1 cell. The statistical analysis was performed using the t test in GraphPad Prism 8. *: *p* < 0.05, ***: *p* < 0.001.

To assess the bioactivity of IL-17B expressed in duckweed, we purified the recombinant protein and performed the following in vitro experiments. The purified protein was incubated with DF-1 cells after 4 h to analyze the expression of immunoadjuvant-related genes including MyD88, NF-κB, IFN-γ, IL-1β, JAK2, and IL-4. In the NF-κB pathway, the expression of MyD88 and NF-κB were significantly higher than those in the control group (Figure 8). In addition, IL-17B stimulated the expression of cytokines (IFN-γ, IL-1β and IL-4) and caused significant upregulation of JAK2 in DF-1 cells.

### 3.3. ChIL17B Transgenic Duckweed Exhibited Potent Immunoadjuvant Activities in Chickens

#### 3.3.1. Enhancement of Humoral Immune Responses

To investigated the potential of chIL-17B transgenic duckweed to be vaccine adjuvant, we used it with a commercial live attenuated IBV vaccine H120 (mucosal vaccine) in chickens. Chickens were randomly divided into four groups: IL17B adjuvant group (IL-17B-H120), vaccine-alone group (PBS-H120), wildtype duckweed control group (ZH0403-H120) and PBS control group (PBS). Except the PBS control group, which was left unvaccinated, the other three groups were orally vaccinated with H120 vaccine. Freeze-dried chIL-17B transgenic duckweed was fed four times during vaccination to chickens in the IL17B adjuvant group. Freeze-dried wildtype duckweed (ZH0403) and PBS were fed as control in the wildtype duckweed control group and the vaccine-alone group respectively. Chickens in all groups were intranasally infected with IBV virus after their final immunization.

**Figure 9:**
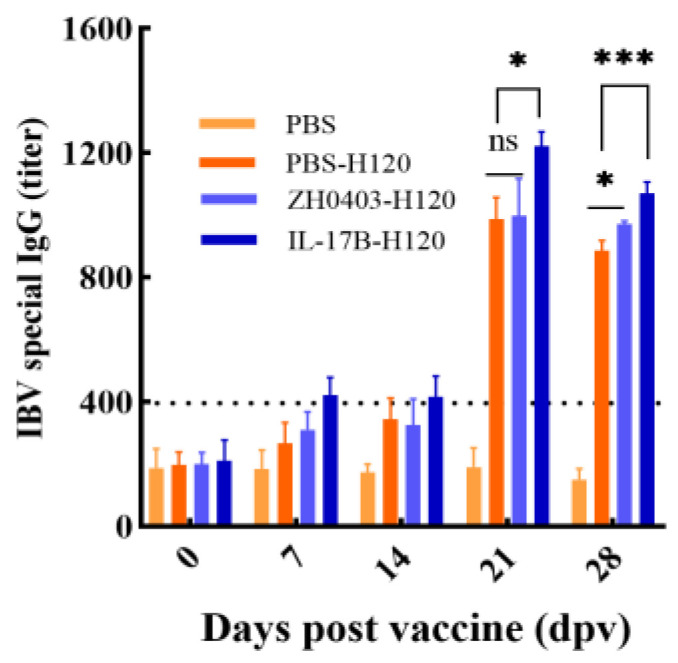
IBV-specific antibodies in the serum detected using ELISA. The IBV-specific antibody levels were measured at 0th, 7th, 14th, 21st, 28th dpv before IBV infection. Values above 396.39 are considered positive. The statistical analysis was performed via one-way ANOVA test. *: *p* < 0.05, ***: *p* < 0.001.

To test the humoral immune responses for chickens, we detected the IBV-specific antibodies in serum via indirect ELISA on the 0th, 7th, 14th, 21st, 28th dpv. As shown in Figure 9, no antibodies were found in the chickens immunized with PBS. Moreover, no IBV-specific antibodies had been detected in any chicken on 0 dpv, indicating that the animals were not infected with bronchitis virus. H120-vaccinated chickens in all groups demonstrated an immune response on the 21st, 28th dpv. In particular, the level of immune response in the IL17B adjuvant group (IL-17B-H120) was markedly higher (*p* < 0.05) than those of the PBS-H120 and ZH0403-H120 groups and these p values decreased below 0.001 on 28th dpv. These results revealed that the IL-17B expressed in duckweed and orally delivered via duckweed can serve as an adjuvant that increases the levels of specific anti-IBV IgGs. Interestingly, the level of immune response in the ZH0403-H120 group (wildtype duckweed control) was also significantly higher (*p* < 0.05) than that of the PBS-H120 group on the 28th dpv.

#### 3.3.2. Mucosal Immune Responses

**Figure 10:**
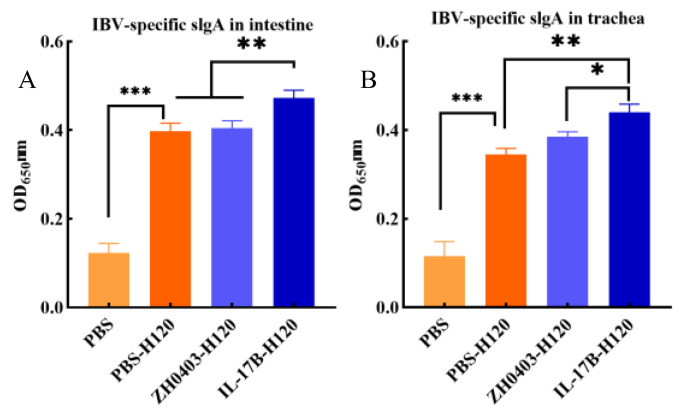
The IBV-specific sIgA titers measured via ELISA under 620 nm wavelength in the intestine (**A**) and trachea (**B**). The error bars represent mean values±SD of each group. One-way ANOVA with Tukey’s test was used for statistical analysis; *: *p* < 0.05; **: *p* < 0.01, ***: *p* < 0.001.

IgA is the main antibody isotype in mucosal secretions and secretory IgA (sIgA), which is polymeric, is the major form of IgA at mucosal sites [29]. Hence, induction of antigen-specific sIgA is the most important indicator of mucosal immune responses after mucosal vaccination. The level of IBV-specific sIgA titers were evaluated via ELISA in tracheas and intestines. Animals with H120 vaccine had an increased mucosal immune response, resulting in the production of a specific sIgA (Figure 10). In particular, the IBV-specific sIgA titers in the IL-17B adjuvant group (IL17B-H120) were significantly higher than those of the PBS-H120 and ZH0403-H120 group in the intestines (*p* < 0.01) (Figure 10A). In the tracheas, the IBV-specific sIgA titers of the IL17B-H120 group were also significantly higher than those of the PBS-H120 (*p* < 0.01) and ZH0403-H120 group (*p* < 0.05) (Figure 10B). Furthermore, the sIgA level was not significantly different between the PBS-H120 group and the ZH0403-H120 group in either the intestine or the trachea. 

#### 3.3.3. Challenges of Protective Immunity against Bronchitis Virus

**Figure 11:**
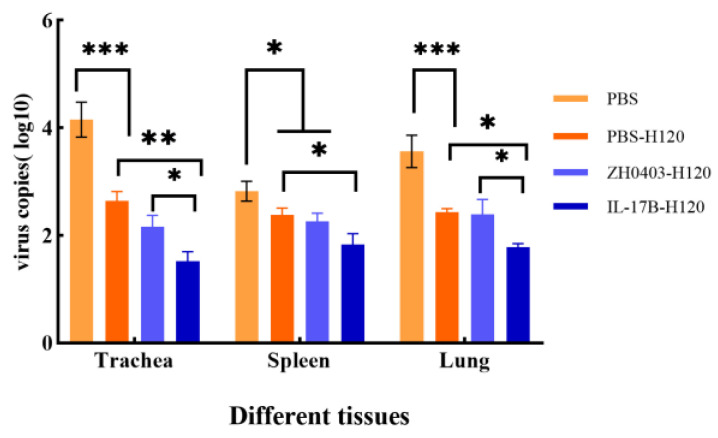
Viral RNA load level in trachea, kidney, and liver determined via absolute quantitative qRT-PCR. One-way ANOVA with Tukey’s test was used for statistical analysis; *: *p* < 0.05; **: *p* < 0.01, ***: *p* < 0.001.

We analyzed whether the oral IL-17B expressed in duckweed can promote protective immunity against bronchitis virus infection. After their final immunization, chickens in all groups were intranasally infected with IBV SCMY-19 virus and then kept for 10 days to examine the viral RNA titers. The virus loads in tissues (trachea, kidney, and lung) were detected via absolute qPCR. As shown in Figure 11, the IBV copies with H120 vaccine groups were lower than those in the PBS control group in all tissues. Importantly, the virus loads in the IL-17B group were significantly lower than those in the PBS-H120 group, *p* < 0.01 in trachea and *p* < 0.05 in spleen and lung. These results illustrate that oral intake of IL17B enhanced H120’s protection against bronchitis virus.

#### 3.3.4. No Effect on Body Weight

**Figure 12:**
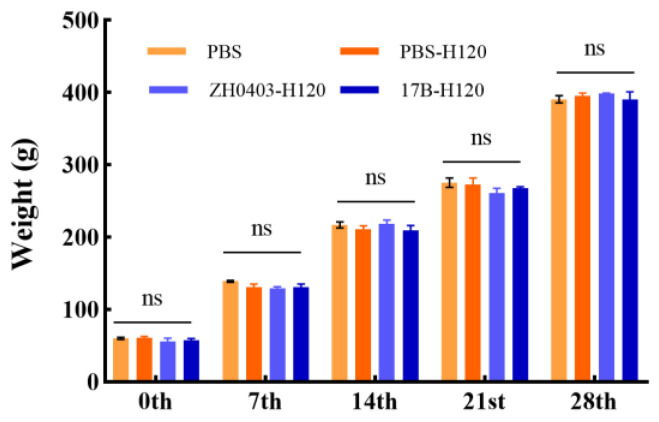
The weight of chickens at 0th, 7th, 14th, 21st, and 28th dpv before IBV infection. PBS: negative control, PBS-H120: positive control, ZH0403-H120: wild plant control, 17B-H120: experimental group. One-way ANOVA with Tukey’s test was used for statistical analysis.

Duckweed, rich in protein content (20–40% dry weight) and essential amino acids, was used for animal feed [12]. Meanwhile, duckweed is an aquatic plant with rapid growth and small size that has been studied by researchers to produce numerous recombinant proteins. In order to evaluate the effect on chickens fed by transgenic duckweed, we weighed the chickens and compared their weights to those of the control group. The weights of the chickens in the four groups were not significantly different from each other on the 0th, 7th, 14th, 21st, and 28th dpv (Figure 12), suggesting the safety of transgenic duckweeds for animals.

## 4. Discussion

Genetic modification of duckweed could be used for plant molecular farming to produce valuable protein. In this study, firstly we report an efficient transformation system in *L. minor* duckweed. Transformation efficiency was always evaluated via GUS or other reporter genes. Here, we compare the transformation efficiency, including transient transformation and stable transformation in DNA and protein level, to previous works on duckweeds and other plants that have reporter gene data (Table 2). As shown in Table 2, our transient transformation efficiency is up to 95%, which is higher than many plants, and comparable with the data of some plants, i.e., *Lemna aequinoctialis*, *Lemna gibba*, Indica rice, and Japonica rice. Similarly, the stable transformation efficiency in DNA level and protein level is also higher than in many plants, while being comparable with some plants, such as *Lemna minor* L. and *Spirodela punctata* 8717. Callus screening took 4 weeks and regeneration took 4 weeks. In short, our system matches or bests the most efficient and rapid systems currently available.

We achieved high transformation efficiency by optimizing both callus induction and the transformation process. Effective callus induction of plants is crucial to molecular farming. In duckweed, the choices of genotypes greatly influence transformation efficiency [25,49]. Previously, we found *L. minor* strain ZH0403 had a high callus induction rate under the T-1 condition in our screening (date not shown), though it took more than 3 months in regeneration. As a result, we chose ZH0403 as the start point and then optimized the culture condition. Many reports found that the rate of callus induction can be significantly increased through optimizing the concentration of phytohormones or medium component in *L. punctata* [26], *L. gibba* [65], and *S. polyrhiza* [66]. According to our data, switching from B5 to MS basal medium and reduction of phytohormone concentration not only increased the callus induction rate of ZH0403 but also reduced its regeneration time from 3 months to 1 month. Although we and some researchers performed procedures based on the original protocol to induce calluses from *L. minor* [67], our transient transformation efficiency (94%) is significant higher than that of Chabra (3.8%) [50] and Canto (59%) [27]. These results suggest that we have chosen an appropriate genotype of *L. minor* on which to perform genetic transformation and this genotype is very suitable for this callus induction protocol. Our work provides a new method to improve the efficiency of callus induction in duckweed, which is culture medium optimization combined with selection of suitable genotypes to start. We also optimized the transformation process by improving the type and dose of screening agent. Many researchers used kanamycin as the selective agent in duckweed, but we utilized G418 as the selective agent. Since monocotyledonous plants such as Zea mays [68], Oryza sativa [69] and *Leighton* [70] usually have a high level of kanamycin tolerance; G418 has been used on some of them as a screening agent for higher efficiency. Furthermore, G418 is generally more toxic than kanamycin and kills untransformed cells more quickly [71]. In our system, 100 mg/L G418 can kill the untransformed cell (Figure 3B), resulting in a high transformation rate (93%). Taken together, appropriate genotype, optimization of culture medium, and the use of high concentration of G418 for selecting evidence success in establishing a highly efficient transformation system. Our work not only provides a duckweed platform for molecular farming, but also provides useful knowledge for plant genetic transformation. 

In this study, we expressed recombinant chIL-17B in duckweed as a form of molecular farming and investigated the potential of chIL-17B transgenic duckweed to be adjuvant for mucosal vaccines in chickens. Our work is the first to produce recombinant chIL-17B in plants and our experiments on DF-1 cells evidenced its biological activity in vitro. Our work is also the first to use transgenic plants expressing cytokine as a vaccine adjuvant. A commercial live attenuated IBV vaccine H120 was orally administrated to chickens with or without chIL17B transgenic duckweed as the adjuvant against IBV infection. The level of IBV-specific antibodies in the IL17B-H120 group was markedly higher (*p* < 0.05) than those in PBS-H120 and ZH0403-H120 (wildtype duckweed control) groups. Moreover, the sIgA level is one of the key factors in estimating oral vaccines. The IBV-specific sIgA in the tracheas and intestines of the IL17B-H120 group was significantly higher than in those of other groups. Another important index in evaluating vaccines is the virus load in organs after infection. The main sites of IBV replication are the trachea, the kidney, and the lung [72]. Comparing the virus loads in these tissues, the IL-17B-H120 group had the lowest values in all groups. Interestingly, the wildtype duckweed control group ZH0403-H120 also had significantly higher antibody titer compared with that of the PBS-H120 group in serum at 28 dpv. Some bioactive compounds in plants including saponins, lectins, glycosides, and flavoids have been studied as candidates for adjuvant formulations [73]. Sergey [74] demonstrated that apiogalacturonanic pectin from *L. minor* duckweed enhanced anti-ovalbumin IgG Abs level in serum when it was used as an oral adjuvant for the ovalbumin antigen. Thus, we suspect that *L. minor* contains some active substances that may increase the production of anti-IBV IgG Abs after vaccination. Taken together, our data demonstrate that IL-17B produced and orally delivered via duckweed can serve as an effective adjuvant to enhance humoral and mucosal antibody response and reduce virus load.

Cytokines expressed in plants are bioencapsulated in plant cells and are finally delivered to the gut. The gut-associated lymphoid tissue (GALT) is the direct target of mucosal vaccines and adjuvants orally administrated [75,76]. So mucosal vaccines against gastrointestinal infections are usually oral vaccines, such as the cholera vaccine, salmonella vaccine, poliovirus vaccine, and rotavirus vaccine [29]. Due to the interconnection between GALT and other mucosal sites, IgA secreting plasma cells can migrate from GALT to other mucosal sites such as the nose, trachea and lung, enabling oral vaccines to promote robust mucosal immunity at effecter sites distant from the gut [77,78]. Currently, more and more efforts have been devoted to develop oral formulation of mucosal vaccines against respiratory infections, such as influenza [79] and COVID-19 [80]. Moreover, for avian mucosal vaccines, such as Newcastle disease vaccine and infectious bronchitis vaccine, administration through the intranasal route or oral route are both recommended [81]. In our work, we observed that chIL17B transgenic duckweed as adjuvants caused significant enhancement of mucosal immunity not only in intestines but also in the trachea. Our data indicate that cytokines expressed in transgenic duckweed have the potential to be developed as adjuvants for mucosal vaccines administrated orally.

Edible plant-based cytokine adjuvants have the advantages of low cost, high efficiency, and convenience. They are cost-effective because they are administrated by feeding directly and thus expensive downstream manufacturing processes are eliminated [3]. In addition, recombinant proteins in lyophilized plants are stable at room temperature and do not require cold chains during storage and transportation [4]. Moreover, they can be used with commercial vaccines directly, able to enhance immune responses of commercial mucosal vaccines in a fast and easy way in the animal breeding industry. Our work suggests a novel strategy to develop mucosal vaccine adjuvants that is strong and safe in a low-cost and efficient way, and also suggests a new application of molecular farming for vaccine adjuvants.

## 5. Conclusions

In summary, we report an efficient transformation system in *L. minor* duckweed with a high transformation efficiency that takes 7–8 weeks from screening calluses to transgenic plants. We expressed chIL-17B using our system and evaluated its biological activity in vitro and its function as a vaccine adjuvant in chickens. This work is the first to investigate oral delivery of cytokines expressed in edible plants as vaccine adjuvants. Our data demonstrate that duckweed-based chIL-17B as an adjuvant effectively enhances systemic and mucosal immune responses, especial mucosal sIgA level at effecter sites, and reduces virus load. Our work suggests cytokines expressed in transgenic duckweed are promising adjuvants for mucosal vaccination through the oral route.

## Figures and Tables

**Table 1:** The composition of callus induction mediums.

Varian	Induction Medium		Subculture Medium		pH	Culture Condition
	Basal Medium	Phytohormones	Basal Medium	Phytohormones		
T-1	B5 + 10 g/L su + 0.35% Ge	2,4-D 45 μM + TDZ 5 μM	B5 + 10 g/L Su + 0.35% Ge	2,4-D10 45 μM + TDZ 5 μM	5.8	16 h day/8 h night
T-2	B5 + 10 g/L su + 0.35% Ge	2,4-D 0.45 μM + 2-IP 5 μM	B5 + 10 g/L Su + 0.35% Ge	2,4-D 1 0.45 μM + 2-IP 5 μM	5.8	16 h day/8 h night
T-3	MS + 30 g/L su + 0.35% Ge	2,4-D 5 μM + TDZ 0.5 μM	MS + 30 g/L Su + 0.35% Ge	2,4-D 1 μM + 6-BA 2 μM	5.6	16 h day/8 h night
T-4	MS + 30 g/L su + 0.35% Ge	2,4-D 4.5 μM + TDZ 0.45 μM	MS + 30 g/L Su + 0.35% Ge	2,4-D 4.5 μM + T DZ 0.45 μM	5.9	24 h night
T-5	MS + 30 g/L su + 0.35% Ge	TDZ 0.45 μM	MS + 30 g/L Su + 0.35% Ge	2,4-D 0.9 μM	5.6	16 h day/8 h night
T-6	SH + 20 g/L gl + 10 g/L So + 10 g/L Ma + 0.35% Ge	2,4-D 22.5 μM + 6-BA 2.2 μM	SH + 20 g/L Gl + 10 g/L Sor + 10 g/L Ma + 0.35% Ge	2,4-D 22.5 μM + 6-BA 2.2 μM	5.6	16 h day/8 h night
T-7	MS + 30 g/L Su + 0.35% Ge	NAA 10 μM + TDZ 0.5 μM	MS + 30 g/L Su + 0.35% Ge	2,4-D 1 μM + 6-BA 2 μM	5.6	16 h day/8 h night

6-BA: 6-Benzyladenine; 2-IP: N6- (2-Isopentenyl) adenine; NAA: 1-Naphthlcetic acid; 2,4-D: 2,4-Dichlorophenoxyacetic; TDZ: thidiazuron; Ge: gellan gum; Su: sucrose; Gl: glucose; Ma: mannitol; So: sorbitol.

**Table 2:** Comparison of transformation efficiency among duckweeds and other plants.

Species	Explant	Gene	Selection Time	Regeneration Time	Callus/Frond Transient Efficiency	Stable Transformation Efficiency in DNA Level	Stable Transformation Efficiency in Protein Level	Reference
*Lemna minor* ZH0403	Callus	GUS	3–4 weeks	4 weeks	95%	88%	86%	This study
*Lemna aequinoctialis* 6002	Callus	GUS	5–6 weeks		94%	-	-	[25]
*Lemna minor* L.	Callus	GUS-M2e	8–10 weeks		-	85%	85%	[15]
*Spirodela punctata* 8717	Frond	GUS	8 weeks		92%	-	80%	[22]
*Wolffia globosa* 5563	Frond	GUS	-	-	-	-	21.8%	[47]
*Lenna gibba* G3	Frond	GUS	-	-	100%	-	17%	[23]
*Spirodela polyrhiza* 5543	Callus	GUS	-	-	-	-	13%	[48]
*Lemna minor* strain ZH0055	Callus	GUS	36 weeks		80%	-	4%	[49]
*Lemna minor*	Callus	GUS	-	-	89%	-	3.8%	[50]
*Lemna minor*	Frond	GUS	9 weeks		30–40%	-	2–6%	[49]
*Spirodela oligorrhiza*	Callus	GFP	4–6 weeks		-	-	0.5–5%	[51]
*Lemna aequinoctialis*	Callus	GFP	10 weeks	4 weeks	49%	-	3%	[52]
*Spirodela punctata*	Callus	GUS	-	-	-	-	2.4%	[22]
*Lemna minor*	Callus	GFP	5 weeks		59%	-	-	[27]
*Wolffia arrhiza*	Callus	Hpt	6–8 weeks	-	-	-	0.2–0.4%	[43]
*Wolffia globosa* 5563	Callus	GUS	4 weeks	-	-	-	0.14%	[47]
Indica rice	Callus/frond	GUS	-	-	99%/49.5%	-	-	[53]
Japonica rice	Callus	GUS	-	-	10–91%	-	-	[54]
*Oryza sativa* L.	Seed	GUS	-	-	-	40%	43%	[55]
Indica rice	Callus	GUS	-	-	-	9.3–23.4%	7–18.9%	[42]
Japonica rice	Callus	GUS	-	-	-	-	0–20%	[56]
Barley	Immature embryo	Hpt	-	-	-	-	4–33%	[57]
Barley	Callus	GFP	-	-	47–76%	-	0.6–4.4%	[58]
Sorghum	Immature embryo	GUS	-	-	41.3%	-	14.2%	[41]
Sorghum	Immature embryo	GUS	-	-	21–63%	-	0.71–10.1%	[59]
Nicotiana tabacum	Leaf	GUS	-	-	-	-	53%	[60]
*Nicotiana tabacum*	Callus	GUS	-	-	-	15%	-	[61]
*Nicotiana tabacum*	Callus	GUS	-	-	-	41.79–53.13%	-	[62]
*Triticum aestivum* L.	Seed	GUS	-	-	-	29–38%	-	[63]
Wheat	Embryo	GUS	-	-	-	68%	41%	[64]

The symbol of “-” represents the data not presented in corresponding papers or patents.

## Data Availability

The data presented in this study are available on request from the corresponding author.
